# Large Hernia of Umbilical Cord Misdiagnosed as Omphalocele

**Published:** 2015-07-01

**Authors:** Syed Waqas Ali, Anwar Arain

**Affiliations:** Department of Paediatric Surgery, National institute of Child Health Karachi, Pakistan

**Dear Sir**

We read with interest case series of “Hernia of umbilical cord: Report of three unusual cases”  [1] and want to mention our own case where a misdiagnosis led to inadequate management resulting in loss of 30cm of small bowel. A 12-hour-old male baby delivered via Caesarian section due to prenatal diagnosis of omphalocele. He was attended by a surgical resident and labelled as omphalocele major and advised mercurochrome paint. Next day, the neonate became lethargic. Abdominal examination showed protrusion of bowel through the umbilicus into an omphalocele like sac with dark maroon discoloration on one side (Fig. 1). There was not abdominal wall defect and umbilicus was normally sited. At the base there was a skin collar of about 2 cm. The patient was immediately prepared for laparotomy with a suspicion of bowel necrosis. On opening the sac, serosanguineous fluid was drained. The part of ileum was necrotic while most of jejunum and ileum were edematous. The incision extended cranially in midline and small bowel was delivered. Grossly necrotic ileum measuring 30cm was excised and end to end anastomosis was done. Malrotation was also present and corrected. Primary closure easily achieved due to bowel resection. The postoperative recovery was uneventful with a follow-up of 6 months. Our case is unique since large sac with hemorrhage was giving appearance of herniated liver causing the condition to be misdiagnosed as omphalocele major. It is mandatory to look carefully in a case of omphalocele for presence of any true abdominal wall defect, umbilical ring, and the base for presence of skin collar so that the condition is not misdiagnosed.

**Figure:**
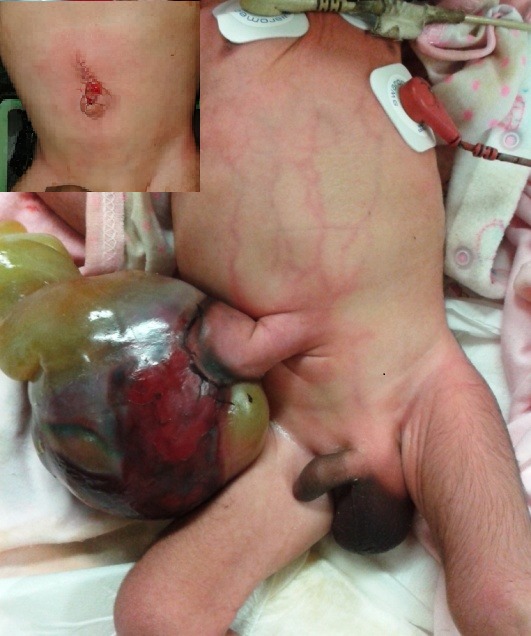
Figure 1: Hernia of umbilical cord with large sac and dark maroon discoloration. Inset showed post-repair abdomen.

## Footnotes

**Source of Support:** Nil

**Conflict of Interest:** None

